# Thomasville, Ga.

**Published:** 1888-04

**Authors:** T. S. Hopkins

**Affiliations:** Thomasville, Ga.


*Editors Atlanta Medical and Surgical Journal:*

DEAR SIRS—Necessity is really at *times* the *mother* of invention, and as *useful* inventions should be made known *pro bono publico*, I send you this. Not very long since a patient was sent to me from a Florida "*resort*," said to be suffering from "*malaria*," that fearful *specter*, which just now seems to haunt our entire continent. I found the patient suffering from *syphilitic* paraplegia. His bowels and bladder were distended to their utmost capacity. After moving the bowels with a copious enema, aided by persistent kneading, I attempted to pass a catheter into the bladder, but failed. He was badly strictured. The catheter passed the first stricture with little trouble, but when it reached the NEXT, in the membranous portion, there was "no go." The point entered the stricture so far as to be firmly grasped by it, but no farther would it go. I was about to give up in despair, when it occurred to me that if I would attach to the end of the catheter the Davidson's syringe, which I had just used to open the bowels, and with my left hand grasp the penis and closely and firmly close the urethra around the catheter, so as to make the bladder a closed sack, I might "aspirate" it. I tried it and was successful, not only once, but every time it was tried.

With a catheter *perforated* in its *point* and a Davidson's syringe, I believe that almost any case of retention of urine can be relieved. Give it a trial.

Very truly yours, T. S. HOPKINS, M. D.

*Thomasville, Ga., January* 26, 1888.

